# Impact of ocean warming and ocean acidification on asexual reproduction and statolith formation of the symbiotic jellyfish *Cotylorhiza tuberculata*

**DOI:** 10.1371/journal.pone.0254983

**Published:** 2021-08-04

**Authors:** Angélica Enrique-Navarro, I. Emma Huertas, Manuel Jesús León Cobo, Laura Prieto

**Affiliations:** Department of Ecology and Coastal Management, Instituto de Ciencias Marinas de Andalucia (CSIC), Puerto Real, Cadiz, Spain; University of Connecticut, UNITED STATES

## Abstract

Ocean acidification and warming are challenging marine organisms and ecosystems around the world. The synergetic effects of these two climate change stressors on jellyfish remain still understudied. Here, we examine the independent and combined effects of these two environmental variables on polyp population dynamics of the Mediterranean jellyfish *Cotylorhiza tuberculata*. An experiment was conducted to examine asexual reproduction by budding and strobilation considering current and ca. 2100 winter (Trial 1, 36 days) and summer (Trial 2, 36 days) conditions under the RCP8.5 (IPCC 2013). In Trial 1, a temperature of 18°C and two pH levels (current: 7.9 and, reduced: 7.7) were tested. Trial 2 considered two temperature levels 24°C and 30°C, under current and reduced acidification conditions (8.0 and 7.7, respectively). Ephyrae size and statolith formation of released ephyrae from polyps exposed to summer temperatures under both acidification treatment was also analyzed. Zooxanthellae density inside the polyps throughout the experiment was measured. *C*. *tuberculata* polyps could cope with the conditions mimicked in all experimental treatments and no significant effect of pH, temperature, or the combination of both variables on the abundance of polyps was observed. At 18°C, strobilation was reduced under high *P*_CO2_ conditions. Under summer treatments (24°C and 30°C), percentage strobilation was very low and several released ephyrae suffered malformations and reduced size, as a consequence of reduced pH and elevated temperatures, separately. The number of statoliths was not affected by pH or temperature, however, bigger statoliths were formed at elevated temperatures (30°C). Finally, zooxanthellae density was not affected by experimental conditions, even if, the duration of the experiment significantly affected symbiont concentration. Our results show that even though polyps of *C*. *tuberculata* would thrive the future worst scenario predicted for the Mediterranean Sea, their capacity to undergo a proper strobilation and to produce healthy ephyrae will be more vulnerable to climate induced environmental conditions, thereby affecting medusae recruitment and, therefore, population dynamics of the species.

## Introduction

The world’s oceans are becoming warmer and more acidic as a consequence of the rapid rise of atmospheric carbon dioxide (CO_2_) concentrations. The ocean mitigates the greenhouse effect by storing excess heat from global warming and by absorbing and storing anthropogenic CO_2_ [1, 2]. However, this contribution is leading to an increase in seawater temperatures and a gradual decrease of pH, known as ocean acidification (OA) [3]. Since preindustrial times the global ocean temperature has increased by 0.5°C and global average pH has declined by 0.1 units [4–6]. By 2100, under a “high CO_2_ emissions” scenario (RCP8.5, [4]), sea surface temperature is predicted to rise between 2.6 and 4.8°C, and seawater pH to be reduced by 0.32.

The effects of OA and ocean warming on marine ecosystems are being extensively studied to assess their consequences on organisms physiology and future populations trends [7]. Major research has been however, focused on calcifying species [8] due to the direct effects of OA on carbonate chemistry, as the concentration of calcium carbonate decreases as does pH [9]. Alternatively, non-calcifying organisms remain understudied [10, 11] even though it has been suggested that at a community level and within the same trophic level, species more tolerant to high CO_2_ might displace others more vulnerable to elevated *P*_CO2_ [12–14]. This tendency, would lead to the proliferation of opportunistic species, such as jellyfish, with a higher resilience to warming and acidification [15]. Therefore, assays combining exposure to changing temperature and *P*_CO2_ in non-calcifying organisms are needed to disentangle biological responses and adaptation to future climate change conditions [16].

During the last two decades, the occurrence of jellyfish blooms has been linked to human-driven ecosystem changes [17] and environmental variations [12, 18]. Many factors have been attributed to influence the abundance of jellyfish, such as overfishing [19], eutrophication [18, 20], temperature increase [21–23] and OA [5, 15, 24].

Scyphozoan jellyfish have a complex bipartite life cycle with alternation of a pelagic sexual stage (medusa) and benthic asexual stage (polyp). Higher temperatures generally leads to increased rates of asexual reproduction of polyps [25]. Yet, the consequences of OA on jellyfish populations remain still diffuse. Despite some early field studies suggested a direct correlation between decreasing seawater pH and abundance of gelatinous zooplankton [26, 27], no direct evidence exists to relate increasing jellyfish blooms and OA [28]. The first laboratory study on the influence of OA on jellyfish polyps showed that survival and asexual reproduction of *Aurelia labiata* polyps were unaffected by experimental conditions [15]. To our knowledge, there are only a few studies focused on the simultaneous effects of warming and acidification on jellyfish polyps using a realistic end-of-the-century climate scenario, and all of them have dealt with cubozoan species [24, 29, 30]. Polyps of the Irukandji jellyfish *Alantina* nr *mordens* were able to cope with warming but, their budding capacity decreased by lowering seawater pH, thereby limiting their possibilities to thrive [24]. On the other hand, polyps of *Carukia barnesi* showed tolerance to extreme conditions [30]. Despite asexual reproduction appears to be independent of the environmental forcing imposed by both stressors, higher respiration and metabolic rates have been reported in response to warming and OA [29]. Similar results regarding asexual reproduction of scyphozoan polyps were obtained when combining reduced pH with low oxygen concentrations [31, 32]. Recent laboratory studies have tested the effects of OA on other jellyfish life stages, from planula larvae [33, 34] to ephyrae [31, 32, 35] and even on adult medusae [36–38]. The differential responses reported seem to be related to the magnitude of the stressors, life stage, and species-specific tolerance limits.

Massive occurrences of jellyfish populations have been considered a threat to Mediterranean planktonic communities [39]. As the Mediterranean Sea is already experiencing the impact of climate change [40], it has become a priority to elucidate how the future expected warming and acidification conditions will influence jellyfish dynamics due to the important repercussions for biodiversity and ecosystem functioning.

In this work, we examine the combined effects of warming and acidification on the polyp population dynamics of the jellyfish *Cotylorhiza tuberculata*, one the most common bloom forming Mediterranean scyphozoans. This species reaches very high abundances during summer in shallow semi-enclosed marine areas, such as Vlyho Bay in Greece [41] and, the Mar Menor coastal lagoon in Spain [42], where annual blooms cause economic losses mainly associated with tourism [43]. *C*. *tuberculata* polyps reproduce asexually by generating free-swimming buds [44], when lateral outgrowth from the parental polyp is released, swims around before attaching and becomes a new polyp. The transition from benthic to pelagic stage also occurs by asexual reproduction, when polyps undergo monodisc-type strobilation, with every single polyp generating one single ephyra [41]. Also, *C*. *tuberculata* harbors endosymbiotic dinoflagellates of the family Symbiodiniaceae [45]. Despite zooxanthellae seem to play a modest role in the polyp stage [46], their presence is indispensable for strobilation [41]. Temperature is known to control survival and asexual reproduction of *C*. *tuberculata* polyps [46]. However, rising temperatures will be accompanied by decreasing pH in the future and therefore, it is critical to investigate the interactive effect of these two stressors to better predict the jellyfish population trends in the future.

Our experiments tested predicted future values of temperature and pH expected to occur in the Mediterranean Sea by the end of the current century under the RCP8.5 [4] scenario. Moreover, we investigated the influence of both stressors on the formation of statoliths, a crystal immersed in the sense organ statocyst, known to play a key role in medusa equilibrium [47, 48].

## Materials and methods

### Ethical statement

This study was carried out in strict accordance with the EU Directive 2010/63 (https://ec.europa.eu/environment/chemicals/lab_animals/interpretation_en.htm). No specific collection permits were required, original animal collection was conducted in accordance with local and national laws and regulations. No animals were harmed for the purpose of this study, except for released ephyrae that were conserved in EtOH for statolith analysis, in this case, the animals’ suffering was minimized. Our field studies did not involve endangered or protected species.

### Experimental design

*C*. *tuberculata* polyps were obtained from laboratory cultures of the Instituto de Ciencias Marinas de Andalucia (ICMAN-CSIC). Polyps used in this study were kept in the laboratory since 2010. Cultures were maintained at 18°C and 12:12 h light: dark cycle in an incubator (IBERCEX F-4) with filtered seawater (0.2 μm). Polyps were fed every three days with rotifers *at libitum* in the dark. Rotifer species used was *Brancionus plicatilis* fed with microalgae *Nannochloropsis gaditana*, *Tetraselmis chuii* and *Isochrysis galbana*. The scyphistomae originated from planula larvae of mature female medusae were collected in the Mar Menor coastal lagoon (37.7702°N, 0.7860°W) located on the Mediterranean southeastern shore of Spain.

A total of 180 arbitrary selected polyps were placed in a set of 12 glass flasks (250 ml volume), each containing 15 polyps, for a 30-days recovery period to allow re-attachment and acclimation to laboratory conditions. Glass flasks were randomly assigned to the different experimental treatments. After recovery, the number of polyps was readjusted (n = 15) before commencing the experiment. Four additional flasks (n = 50), one per treatment, were used to assess zooxanthellae density inside the polyps.

**Fig 1:**
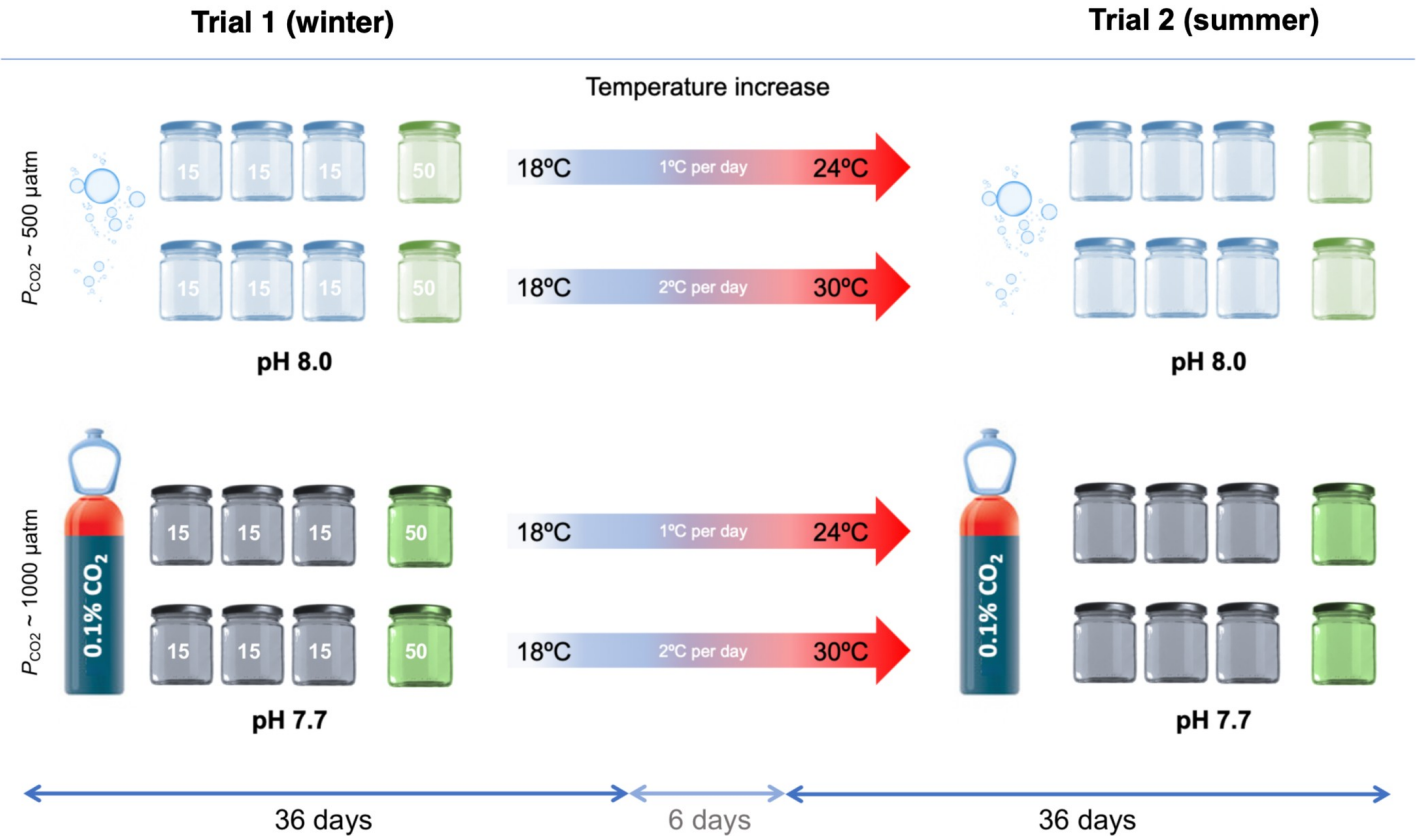
Experimental design. Trial 1 mimicking current and future winter conditions and Trial 2 mimicking current and future summer conditions. Light blue flasks correspond to *P*_CO2_ ~ 500 ppm treatments and grey flasks to *P*_CO2_ ~ 1000 ppm treatments. Additional green flasks were used to study zooxanthellae density inside *Cotylorhiza tuberculata* polyps.

Four experimental treatments were used to mimic four different climate change scenarios (Fig 1): Treatment (1) mimicked ‘present-day temperature and *P*_CO2_’, from winter (18°C) to summer (24°C) temperatures and *P*_CO2_ ~ 500 ppm. Treatment (2) considered RCP8.5 temperature only, a ‘future warmer scenario’ with temperatures ranging from winter conditions of 18°C to elevated summer temperatures of 30°C and *P*_CO2_ ~ 500 ppm. Treatment (3) RCP8.5 *P*_CO2_ only, a ‘future acidified scenario’ from 18°C to 24°C and *P*_CO2_ ~ 1000 ppm. Treatment (4) combined RCP8.5 temperature and *P*_CO2_ considering a ‘future warmer and acidified scenario’ from 18°C to 30°C and *P*_CO2_ ~ 1000 ppm. Temperature and *P*_CO2_ used in the experiment were chosen in accordance with *C*. *tuberculata* polyps natural ranges in the Mar Menor coastal lagoon [49]. Where temperature ranges from 14°C to 18°C in winter, and from 24°C to 26°C in summer. Mean *P*_CO2_ levels measured in winter in the lagoon, were ~ 580 ppm in winter and, ~ 470 ppm in summer.

Two successive trials were done. Trial 1 (T1), mimicked present-day and future end-of-century (RCP8.5 [4]) winter conditions. T1 was first conducted to examine polyp asexual reproduction by budding and strobilation over 18°C and two pH levels, 7.96 (treatments 1 and 2) and 7.69 (treatments 3 and 4). Afterward, T1 glass flasks were used to perform Trial 2 (T2), aimed at assessing rates of asexual reproduction by budding, strobilation, and formation of statoliths in newly metamorphosed ephyrae under current and future summer scenarios (T: 24°C and 30°C; pH: 7.99–8.01 and 7.71–7.73). T2 was also conducted for 36 days. The period of temperature increases from T1 to T2 lasted for 6 days. Change to the summer temperature treatments was applied by a gradual increase of 1°C per day for those flasks that changed from 18°C to 24°C (treatments 1 and 3); and 2°C per day (1°C every 12 hours) for temperature transition from 18°C to 30°C (treatments 2 and 4) (Fig 1). The number of polyps inside the glass flasks was not manipulated between T1 and T2 to mimic polyp population dynamics.

A pre-experiment was conducted to establish the time interval for seawater renewal during the experiments (S1 Fig and S1 Table: in S1 File).

### Experimental set-up

Experiment was conducted in controlled temperature chambers and under a photoperiod of 12:12 h to allow photosynthesis to proceed in zooxanthellae. The carbonate chemistry of seawater was manipulated using a commercial gas mixture bottle containing 1000 ppm of CO_2_ (B-50 Alphagaz As-1, AirLiquide) to achieve the desired future pH values. For the current pH treatment, seawater was also bubbled with ambient air (~ 500 ppm). During bubbling, matrix tanks were sealed to maintain *P*_CO2_ at the required levels and monitored continuously using a 780 pH Meter (Metrohm) previously calibrated with a SWS scale buffer solution [50]. Temperature inside the chambers was monitored every 30 min with a data logger thermometer (EL-USB-TP-LCD+, LASCAR Electronics). Photon flux density was 360 μmol quanta m^−2^ s^−1^. Polyps were fed with rotifers for 2 h every third day. During feeding, flasks stoppers were loosely placed to minimize gas exchange. After feeding, each flask was emptied of water and wholly refilled with seawater of the appropriate temperature and pH. The stoppers were hermetically closed to prevent air bubbles and minimize changes in seawater carbonate chemistry. Finally, each glass flask was returned to its respective thermal conditions.

### Analysis of carbonate chemistry

Every third day, samples were collected from pre-treated seawater for measurements and analysis of temperature, pH, salinity, total alkalinity (*A*_T_), dissolved oxygen (DO), and nitrate and silicate concentrations (Table 1). *P*_CO2_ was subsequently calculated using the on-line software CO2SYS [51] by introducing the measured parameters. Water temperature and DO were monitored with a previously calibrated multi-probe YSI-6920v2 (Yellow Springs, Ohio, USA). Seawater salinity was 38, corresponding to an average value of Mediterranean waters. *A*_T_ was determined by potentiometric titration using a Metrohm 794 Titroprocessor and Fixanal (0.5 mol l^-1^ of HCl) as titrant [52]. pH was measured spectrophotometrically [53] using m-cresol purple as indicator, and consequently, values were expressed in total scale. Nitrate and silicate were obtained following the techniques described by [54].

**Table 1:** Temperature (T), pH (pH_T25_), Dissolved Oxygen (DO), total alkalinity (A_T_) and *P*_CO2_ in the six experimental treatments.

Trial	T1		T2			
**T**	18°C (0.005)		24.2°C (0.015)		30.1°C (0.021)	
**pH**	7.96	7.69	7.99	7.71	8.01	7.73
	(0.03)	(0.02)	(0.02)	(0.01)	(0.02)	(0.03)
**DO (mg l**^**-1**^**)**	7.41	7.58	7.01	7.13	6.54	6.63
	(0.16)	(0.24)	(0.12)	(0.11)	(0.08)	(0.18)
***A***_**T**_ **(μmol kg**^**-1**^**)**	2491.81	2498.9	2516.81	2507.9	2509.21	2507.9
	(13.11)	(11.49)	(13.89)	(20.49)	(16.07)	(20.49)
***P***_**CO2**_ **(μatm)**	535.79	1084	495.63	1021	499.12	1003
	(41.77)	(91.51)	(22.20)	(41.82)	(15.20)	(77.82)

Concentration of nitrate: 23.03 (0.01) μM; and silicate: 8.69 (0.08) μM.

Mean (SEM) *n* = 4 per day and treatment.

### Polyp growth and asexual reproduction by budding

Polyps were counted and examined for survival, asexual reproduction by budding and strobilation every third day during feeding and before water renewal. Ephyrae were removed, preserved in 100% EtOH, and stored in a freezer (-20°C) until statolith analyses. Every detached polyp or free-swimming bud was carefully recovered to be handed back to their respective flask after water renewal. Water within each glass flask was replaced by seawater at the proper temperature and *P*_CO2_ treatment.

The daily increase in number of polyps was calculated as suggested by [55]:

$$Daily\;Budding\;Rate=\frac{\left(N_{final}-N_{initial}\right)}{\left(N_{initial}\right)}\times Total\;Days^{-1}$$

where *N*_*initial*_ is the number of polyps at the beginning of the experiment and *N*_*final*_, the number of polyps at the end of the experiment, and *Total Days* is the duration of the experiments. The daily budding rate (DBR) was expressed as percentage. Mortality and strobilation rates of polyps were also expressed as percentage of the total number of polyps present on a given experimental day.

### Statolith analyses

Ephyrae released at the end of T2 (from day 24 to 36) were chosen for statolith analyses, as those polyps were exposed to elevated temperatures and high *P*_CO2_ much longer. Statoliths of 13 ephyrae with ages ranging from 1 to 3 days were analyzed. Three ephyrae from the four T2 treatments: 24°C and pH 7.99 (Present-day), 24°C and pH 7.71 (RCP8.5 pH only), 30°C and pH 8.01 (RCP8.5 Temperature only) and 30°C and pH 7.73 (RCP8.5 Temperature and pH). Only those well formed ephyrae with 8 arms and similar size were used for statolith analyses.

**Fig 2:**
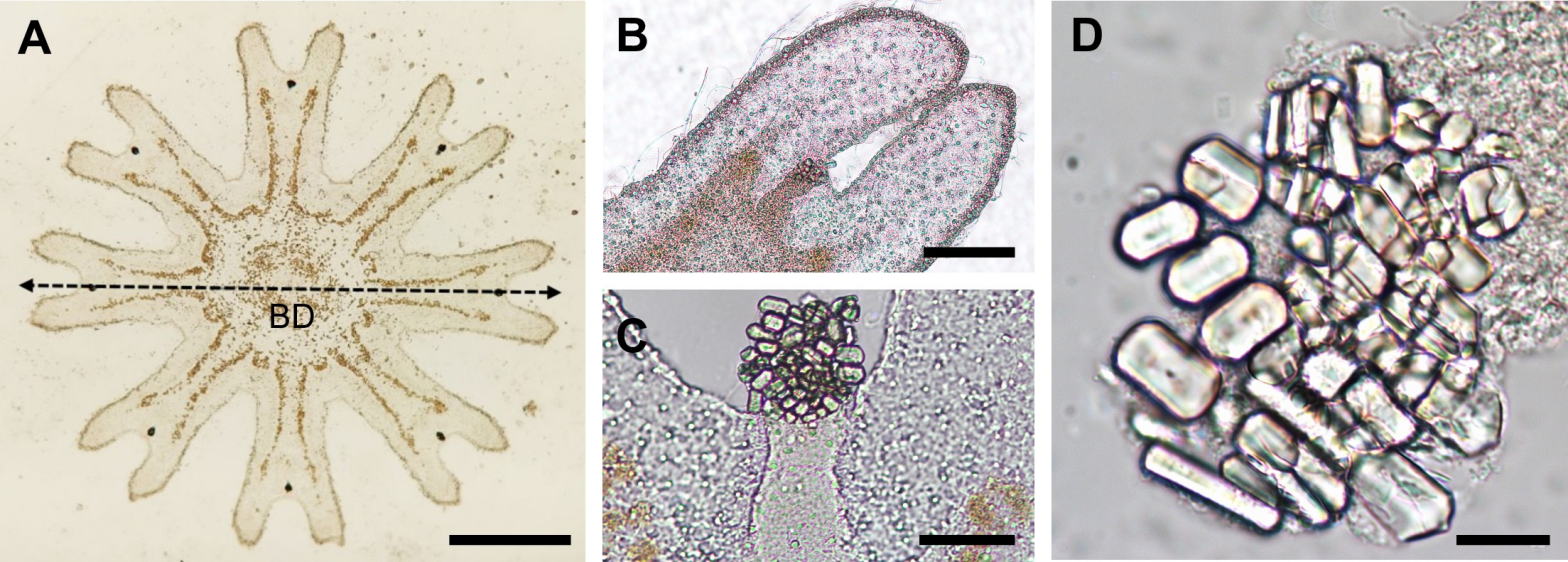
*Cotylorhiza tuberculata* ephyrae and statoliths measurements. A. Ephyrae of *Cotylorhiza tuberculata*. *BD* body diameter. Scale bar = 500 μm. B. Ephyrae marginal lappets and rhopalium. Scale bar = 100 μm. C. Rhopalium with statocyst containing statoliths inside. Scale bar = 50 μm. D. Statoliths. Scale bar = 20 μm.

The total body diameter of the 13 well-formed ephyrae, as well as the diameter of the malformed ephyrae was measured under optical microscope at 5x magnification (Fig 2A). The number of statoliths inside three ephyrae rhopalium was counted (Fig 2B and 2C). The length and width of all statoliths in those three rhopalia were measured on a computer screen using AxioVisionLE software at 100x magnification (Fig 2D). The volume of each statolith was indirectly calculated from measurements of length and width, considering the statolith shape as a hexagonal prism [15]. As strobilation did not occur in all flasks, ephyrae were released from different polyps but not always reared from different flasks (See ‘Statistical analysis’ section).

Additionally, the malformed ephyrae that were not used for statolith analyses were counted and their diameter measured. Ephyrae symmetry was evaluated by observation of the lack of some arm, the lack of some rhopalium, and the presence or absence of statoliths inside their rhopalium.

### Zooxanthellae presence

A set of four additional glass flasks (one per treatment, see Fig 1) each containing 50 polyps, were maintained in parallel to the main experiments under the same temperature and pH treatments, as well as, light regime, water renewal, and feeding conditions previously described for T1 and T2. Every six days, three randomly chosen polyps (*n* = 122) from each treatment were taken using a pipette, mounted on a glass slide, and covered with a coverslip to be examined for the presence of symbionts under optical microscopy. Polyps were photographed and zooxanthellae inside the polyps were counted. Counts were done by randomly locating three 250 μm^2^ squares and counting zooxanthellae inside the squares. Mean zooxanthellae density was obtained from the mean number of zooxanthellae present 250 μm^2^, and the density of zooxanthellae per mm^-2^ was calculated.

### Statistical analyses

Physic-chemical data from three replicate glass flasks in each treatment were daily averaged. In order to assess how different temperature and pH levels affected the asexual reproduction of *C*. *tuberculata* polyps, we estimated different generalized estimating equations models (GEEGLM) by means of the R package “geepack” [56]. A Poisson distribution was used to model the error structure. As the experiment consisted of two dependent trials, the experimental treatments were tested in two different models. The model specifications were as follows: The number of polyps (Final Survivors—Starting polyps) as the response variable. The explanatory variables for trial 1 model were pH treatment, the number of days since the experiment started, and the density of polyps present in the experimental flasks each day. Trial 2 model considered pH, temperature, and the combination of both variables, as well as the number of days since the experiment started, and the density of polyps present in the experimental flasks each day, as explanatory variables.

A mixed-effects model approach using the R package lme4 [57] with the function *lmer* was used to test for differences in the number and volume of statoliths (response variables) using pH and temperature as fixed factors. Individual ephyrae and rearing flask were designated as random effects to control for statocyst being analyzed from the same polyp and different polyps rearing from the same flask. However, not all replicate flasks are represented in the analysis due to the lack of strobilation in some flasks, therefore data analysis must be interpreted carefully. Furthermore, the day of ephyrae release was not considered in the model, as strobilation occurred at the end of T2, from day 24 to 36. As the diameter of malformed ephyrae was also measured, the same model was used to evaluate the effects of temperature and pH in the diameter of all measured ephyrae (*n* = 24), the rearing flask was designated as random effect.

Zooxanthellae density within the polyps was also tested by using a mixed-effects model lme4 [57] to study the effects of pH, temperature and time of experiment on the density of symbionts. Glass flask were designated as random effects to control for polyps being obtained from the same glass flask.

For all tested effects on different response variables, a range of models were investigated to assess the model of best fit by using Akaike’s information criterion (AIC). Data were checked for normality and homoscedasticity using standardized residual and Q-Q plots.

## Results

### Polyp survival and asexual reproduction

**Fig 3:**
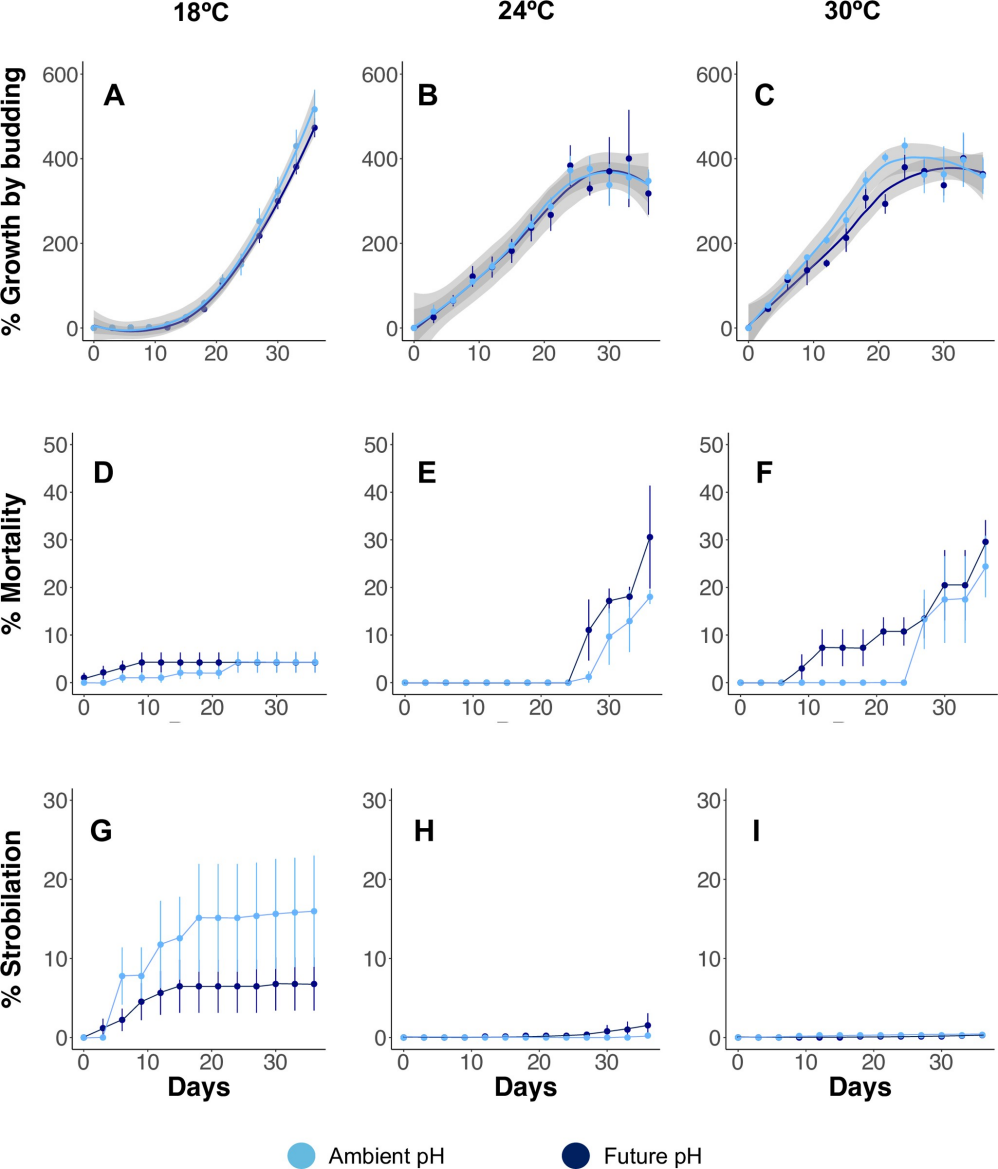
Population growth by budding, mortality and, strobilation of *Cotylorhiza tuberculata* polyps under tested temperatures (18, 24 and 30°C) and pH levels (ambient and future). A, B, C. Percentage of growth of polyps by asexual reproduction (budding). D, E, F. Accumulate percentage of polyp mortality. G, H, I. Accumulate percentage of strobilation. Light blue for ambient pH conditions (*P*_CO2_ ~ 500 ppm) and dark blue for future pH conditions (*P*_CO2_ ~ 1000 ppm). Error bars are standard deviation. Grey bands are standard error.

During T1, at 18°C and current and future OA conditions (pH 7.94 and 7.69, respectively), polyps reproduced by budding from day 12. A delay period prior to the onset of budding was observed at the beginning of the experiment (Fig 3A) then, polyp population grew exponentially. Daily budding rates of the active period of budding with the delay period omitted (from day 12 to day 36) were similar at ambient and future pH treatments, 8.00% (SD = 4.80) and 7.26% (SD = 4.16), respectively. Results of the GEEGLM (Table 2) showed that the asexual reproduction of trial 1 polyps, was not affected by pH (*p* = 0.249). However, the differences in the number of polyps observed through the trial were a consequence of the polyp density (*p* < 0.001) present in each flask at different times and the duration of the experiment (*p* < 0.001). The number of mortalities and surviving polyps at the end of the trial was similar between pH treatments (Fig 3D and Table 3).

**Table 2:** Generalized estimating equations model (GEEGLM) and statistical significance of the fixed effects in explaining the variation of the number of polyps.

	Variable N° Polyps	X^2^	*df*	*P*
Trial 1	pH	2.1	1	0.249
	Polyp density	201.9	1	<0.001***
	Day	15.1	1	<0.001***
Trial 2	pH	0.84	1	0.358
	Temperature	0.63	1	0.426
	pH x Temperature	3.11	1	0.078
	Polyp density	9.59	1	0.002*
	Day	25.80	1	<0.001***

Chisq = X^2^, *df* = degrees of freedom.

*p < 0.05,

*** *p* < 0.001.

**Table 3:** Number of polyps, number of dead polyps and number of released ephyrae counted at the beginning and at the end of the experiment.

Treatment	Replicate	Day	Polyps	Dead polyps	Ephyrae
**18°C, pH 7.96**	R1	1	15	1	3
		36	89		
	R2	1	15	4	4
		36	60		
	R3	1	15	0	10
		36	104		
	R4	1	15	1	0
		36	95		
	R5	1	15	0	0
		36	102		
	R6	1	15	0	3
		36	105		
**18°C, pH 7.69**	R1	1	15	1	2
		36	76		
	R2	1	15	0	0
		36	87		
	R3	1	15	0	2
		36	94		
	R4	1	15	0	0
		36	76		
	R5	1	15	1	0
		36	89		
	R6	1	15	1	3
		36	94		
**24°C, pH 7.99**	R1	1	112	92	1
		36	509		
	R2	1	106	99	2
		36	520		
	R3	1	146	112	1
		36	581		
**24°C, pH 7.71**	R1	1	114	101	0
		36	477		
	R2	1	99	334	0
		36	502		
	R3	1	106	110	20
		36	438		
**30°C, pH 8.01**	R4	1	127	89	4
		36	651		
	R5	1	124	171	0
		36	611		
	R6	1	154	254	0
		36	577		
**30°C, pH 7.73**	R4	1	110	210	1
		36	465		
	R5	1	120	167	2
		36	640		
	R6	1	141	92	2
		36	613		

At 18°C pH 7.96, 18°C pH 7.69, 24°C pH 7.99, 24°C pH 7.71, 30°C pH 8.01, 30°C pH 7.73.

Polyp population continued reproducing by budding during T2, after temperature increase from 18°C to 24 and 30°C (Fig 3B and 3C). GEEGLM revealed no significant effects of pH (*p* = 0.358), temperature (*p* = 0.426), or the combination of both variables (*p* = 0.078) in the asexual reproduction of polyps. The differences in the number of polyps presented through the second trial were also a consequence of the polyp density (*p* = 0.002) and the duration of the experiment (*p* < 0.001). Budding rate was similar at both summer temperatures (24°C and 30°), as well as under ambient and future pH. DBR was 12.44% (SD = 3.18) under 24°C and pH 7.99, and 13.43% (SD = 2.76) under 24°C and pH 7.71. At 30°C, DBR was 14.23% (SD = 4.63) and 14.17% (SD = 3.68) under pH 8.01 and 7.73, respectively. Polyp population at the end of T2 at 24°C had increased by 347.65% (SD = 46.68) and 346.26% (SD = 52.75) at pH 7.99 and pH 7.71, respectively (Fig 3B and Table 3). Polyps started to die by day 27 at both pH conditions (Fig 3E) and accumulate percentage of mortality after 36 days at 24°C was 18.02% (SD = 2.58) and 30.57% (SD = 18.72) under ambient and future pH, respectively (Fig 3E). At the end of T2, 0.25% (SD = 0.11) and 1.53% (SD = 2.66) of the polyps underwent strobilation under current and future pH conditions (Fig 3H). At predicted temperature of 30°C, polyp population growth by budding was similar at under current and future pH, by 360% (SD = 74.56) and 363% (SD = 60.68), respectively (Fig 3C and Table 3). First polyp losses occurred early under future OA conditions (day 9) in comparison with the current conditions (day 27). However, mortality and strobilation at the end of the experiment was similar under both pH treatments (Fig 3F and 3I and Table 3). At the end of the summer experiment, some of the polyps at 30°C that had started the strobilation process died before releasing ephyrae.

### Statolith formation

**Fig 4:**
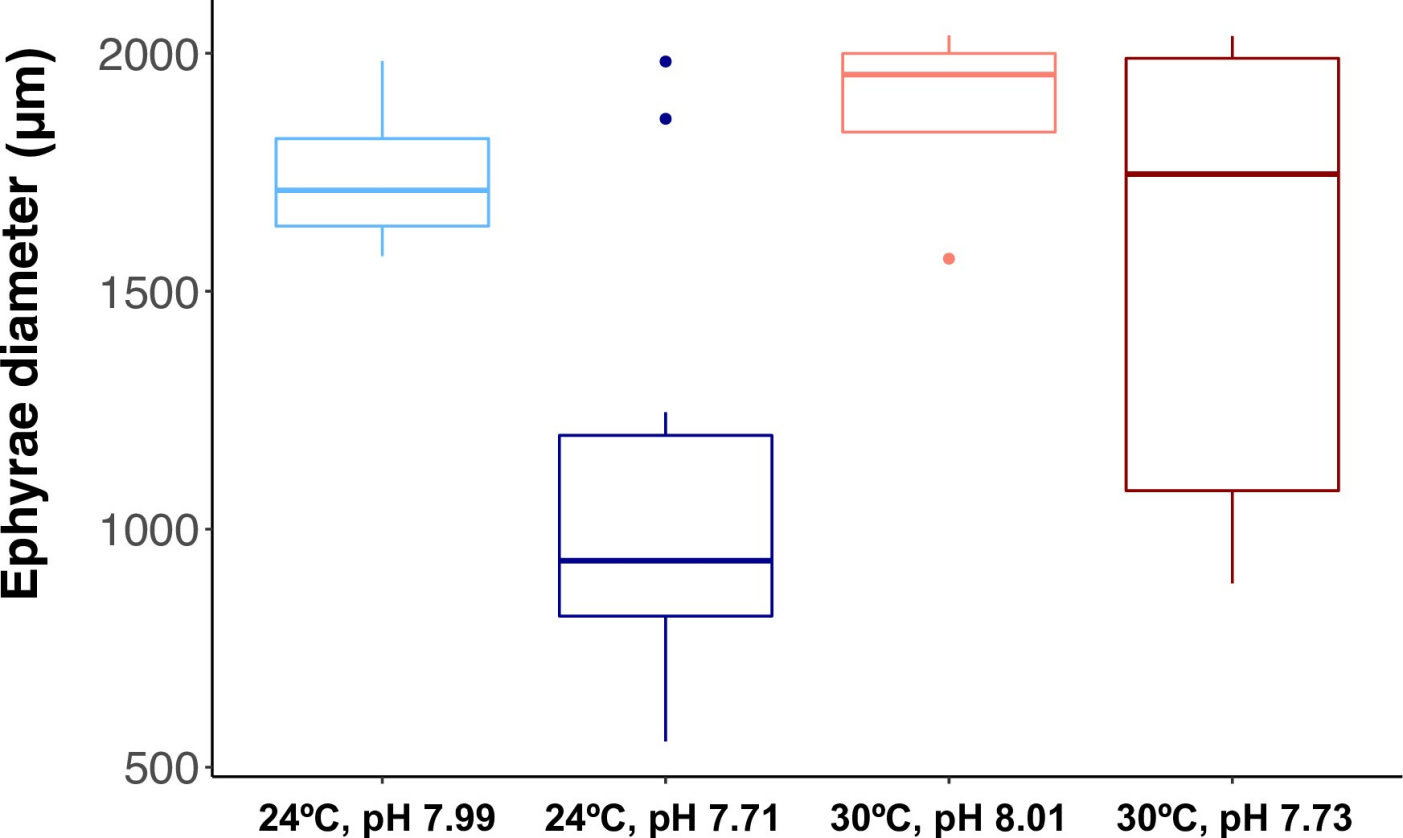
Diameter of ephyrae released under the different temperature (24°C and 30°C) and pH (ambient and future) treatments. Light blue for 24°C and pH 7.99, dark blue for 24°C and pH 7.71, light red for 30°C and pH 8.01 and dark red for 30°C and pH 7.73. Error bars represent standard deviation (SD).

The diameter of the 13 ephyrae used for statolith analyses ranged from 965.99 μm to 2037.57 μm, as ephyrae age ranged from 1 to 3 days old. Ephyrae size was not correlated with the number (*R*^2^ = 0.3527) nor the volume (*R*^2^ = 0.2206) of statoliths (S2 Fig: in S1 File), at least, in well-formed ephyrae (*n* = 13). When comparing all measured ephyrae (*n* = 24), including those presenting malformations, the mixed model (Table 4) revealed significant differences in ephyrae diameter due to temperature (*p* = 0.0432) and pH (*p* = 0.0031). However, no significant effects of the combination of pH and temperature (*p* = 0.3490) were found. Mean ephyrae diameter was higher under ambient pH treatments at both temperatures (Fig 4), at 24°C mean diameter was 1745.51 μm (SD = 177.39) and at 30°C, 1879.05 μm (SD = 212.28). Under future pH mean diameter at 24°C was 1079.37 μm (SD = 433.80) and at 30°C, 1547.84 μm (SD = 531.27).

**Table 4:** Summary of results of the linear mixed-models analysis of the ephyrae diameter (*n* = 24), number of statoliths (*n* = 13) and statolith volume (*n* = 13).

	Fixed factors	*df*	X^2^	*P*
**Ephyra diameter**	Temperature	1	4.09	0.0432 *
	pH	1	8.77	0.0031 **
	Temperature × pH	1	0.88	0.3490
**N. of statoliths**	Temperature	1	0.9786	0.3225
	pH	1	0.0172	0.8955
	Temperature × pH	1	0.1565	0.6924
**Statoliths volume (μm**^**3**^**)**	Temperature	1	4.8399	0.0278 *
	pH	1	1.6273	0.2020
	Temperature × pH	1	0.0522	0.8192

Chisq = X^2^, *df* = degrees of freedom.

*p < 0.05,

*** *p* < 0.001.

**Fig 5:**
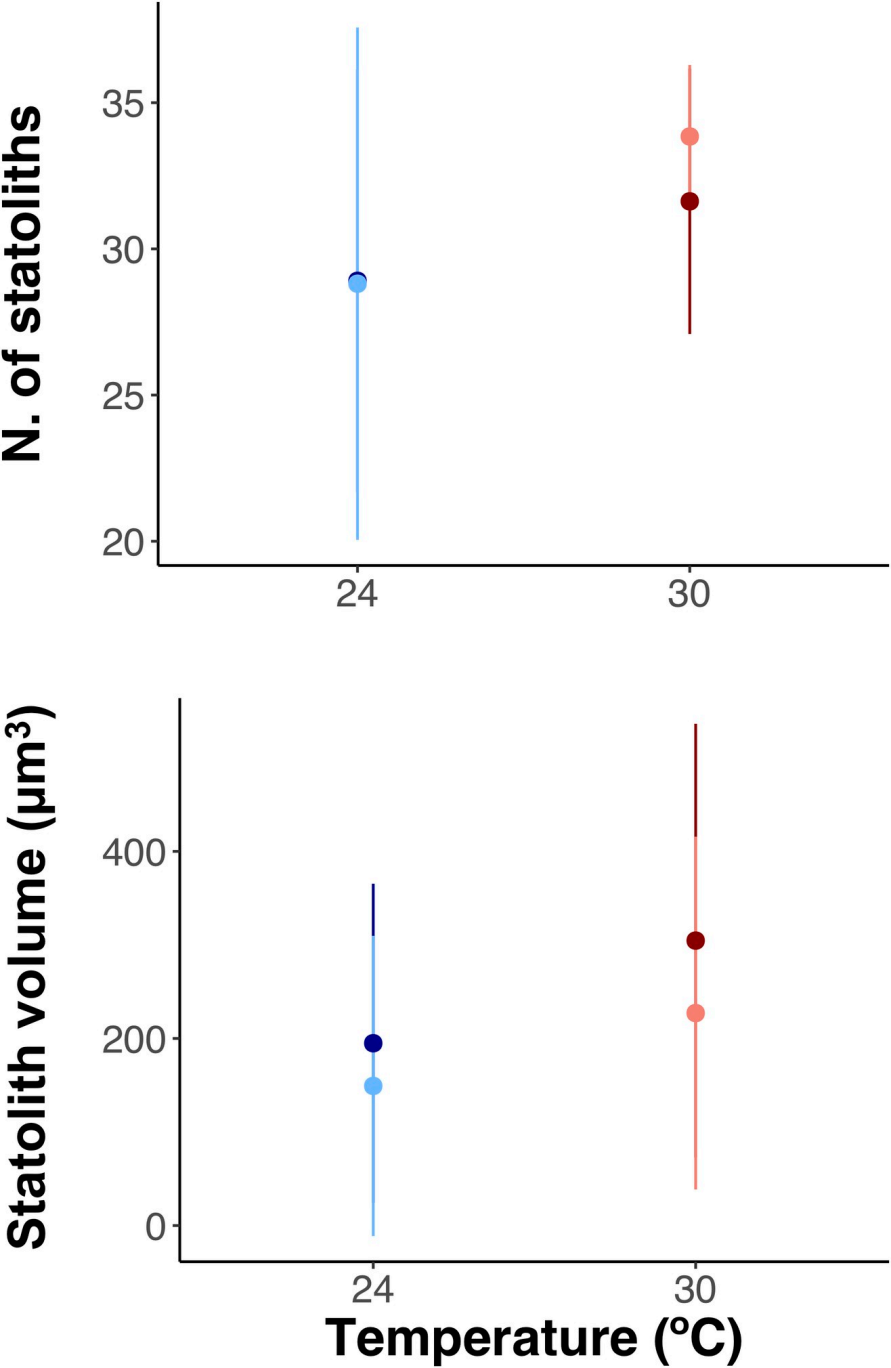
Number of statoliths per rhopalium (A) and volume of statoliths (μm^3^) (B) under the different temperature (24°C and 30°C) and pH (ambient and future) treatments. Light blue for 24°C and pH 7.99, dark blue for 24°C and pH 7.71, light red for 30°C and pH 8.01 and dark red for 30°C and pH 7.73. Error bars represent standard deviation (SD).

The mean number of statoliths per rhopalium (Fig 5A) at 24°C was 26.94 (SD = 7.89) under ambient pH and 28.19 (SD = 5.47) under future pH. At 30°C the number of statoliths were 33.81 (SD = 1.13) and 31.09 (SD = 4.91) under present and future pH, respectively. The results of the mixed-model (Table 4) revealed no significant effects of pH (*p* = 0.8955) and temperature (*p* = 0.3225) or the combination of pH and temperature (*p* = 0.6924) on the number of statoliths per rhopalium.

The volume of the statoliths was affected by temperature (*p* = 0.0278), but pH or the combination of pH and temperature (*p* = 0.2020, *p* = 0.8192, respectively) did not significantly affect statolith size (Table 4). The mean volume of statoliths was smaller at 24°C than 30°C independently of the pH level (Fig 5B). At 24°C and pH 7.99 mean volume of statoliths was 134.99 μm^3^ (SD = 86.18), and 172.77 μm^3^ (SD = 85.15) under pH 7.71. At 30°C the statoliths were bigger under both pH levels than those of 24°C, 226.42 μm^3^ (SD = 64.38) under pH 8.01, and 299.09 μm^3^ (SD = 72.13) under pH 7.73.

**Fig 6:**
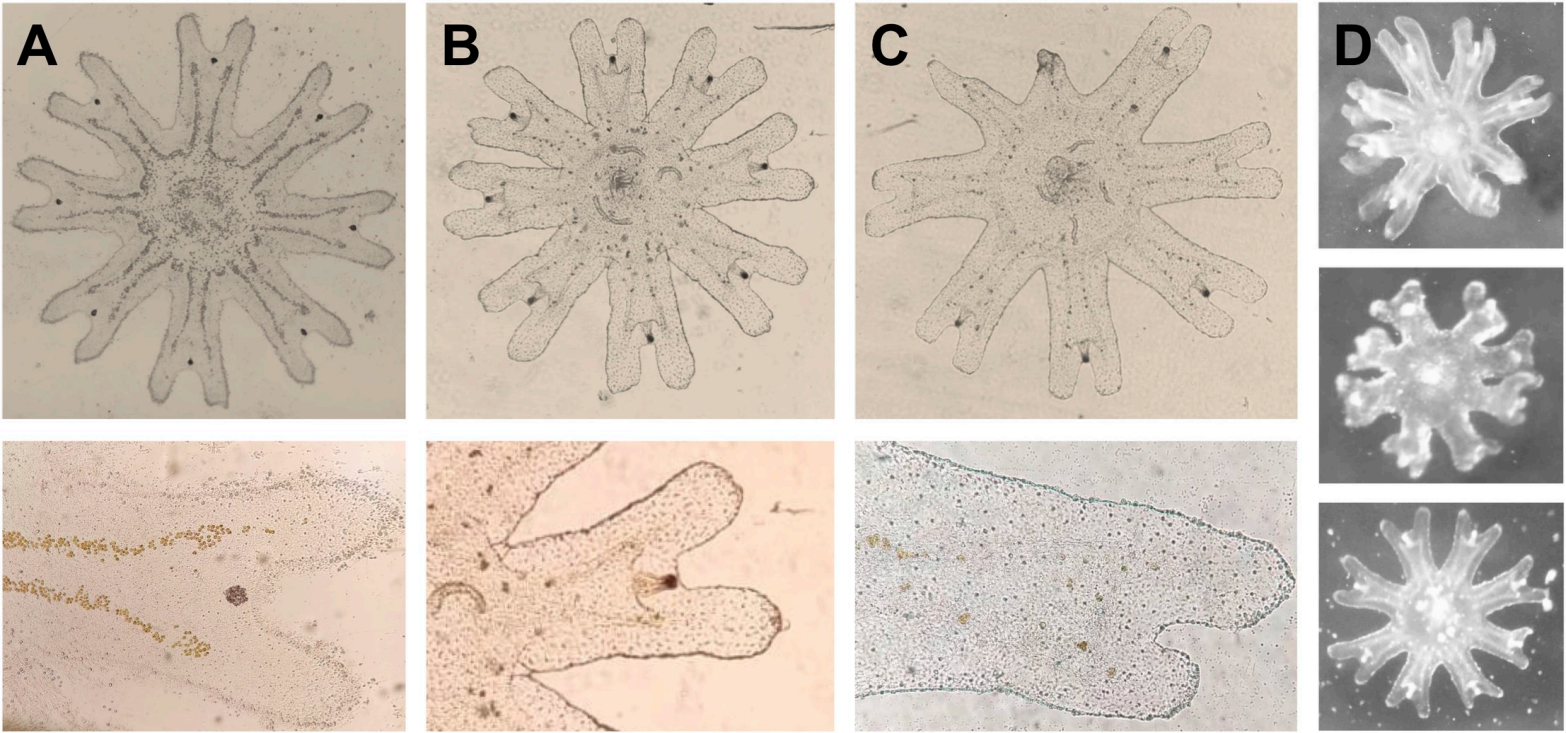
Malformed ephyrae released at the end of the experiment 2. A. Ephyra from the ‘Present day’ treatment at 24°C and pH 7.99 without malformations. B. Malformed ephyra from ‘RCP8.5 temperature only’ treatment at 30°C and pH 8.01. Note the irregular distribution of zooxanthellae and the lack of crystals inside the rhopalium. C. Malformed ephyra from ‘RCP8.5 temperature and pH’ treatment at 30°C and pH 7.73. Note the low zooxanthellae content and the lack of rhopalium. D. Malformed ephyrae from ‘RCP8.5 pH only’ treatment at 24°C and pH 7.71.

Malformed ephyrae were released at the end of the experiment in those treatments that simulated future temperature or acidification conditions, or the combination of both variables (Fig 6 and Table 5). No malformations were detected in the ‘Present day’ treatment (24°C and pH 7.99). Only one malformed ephyra was released in the elevated temperature treatment (30°C), and two irregular ephyrae lacking some arm, rhopalium and statoliths were released under the combination of low pH and high temperature. However, 9 ephyrae from polyps exposed to low pH under 24°C, suffered some kind of malformation, two of them lacked some arm, seven of them did not develop a proper rhopalium and nine of them contained a very low number (1–4 statoliths per rhopalium) of statoliths inside their statocysts. Mean diameter of well-developed ephyrae was 1705 μm (SD = 389) independently of the treatment, the size of the malformed ephyrae was generally lower 1085 μm (SD = 421).

**Table 5:** Number of ephyrae analyzed per treatment, malformed ephyrae and the different kinds of malformations observed (lack of arms, lack of well-developed rhopalium and lack of statoliths inside the statocyst).

Treatment	N. of ephyrae	Malformed ephyrae	Lack of arms	Lack of developed rhopalium	Lack of statoliths inside the statocyst
Present day	4	0	0	0	0
RCP8.5 Temperature only	4	1	0	0	1
RCP8.5 pH only	12	9	3	7	9
RCP8.5 Temperature and pH	5	2	2	2	2

### Zooxanthellae presence

**Fig 7:**
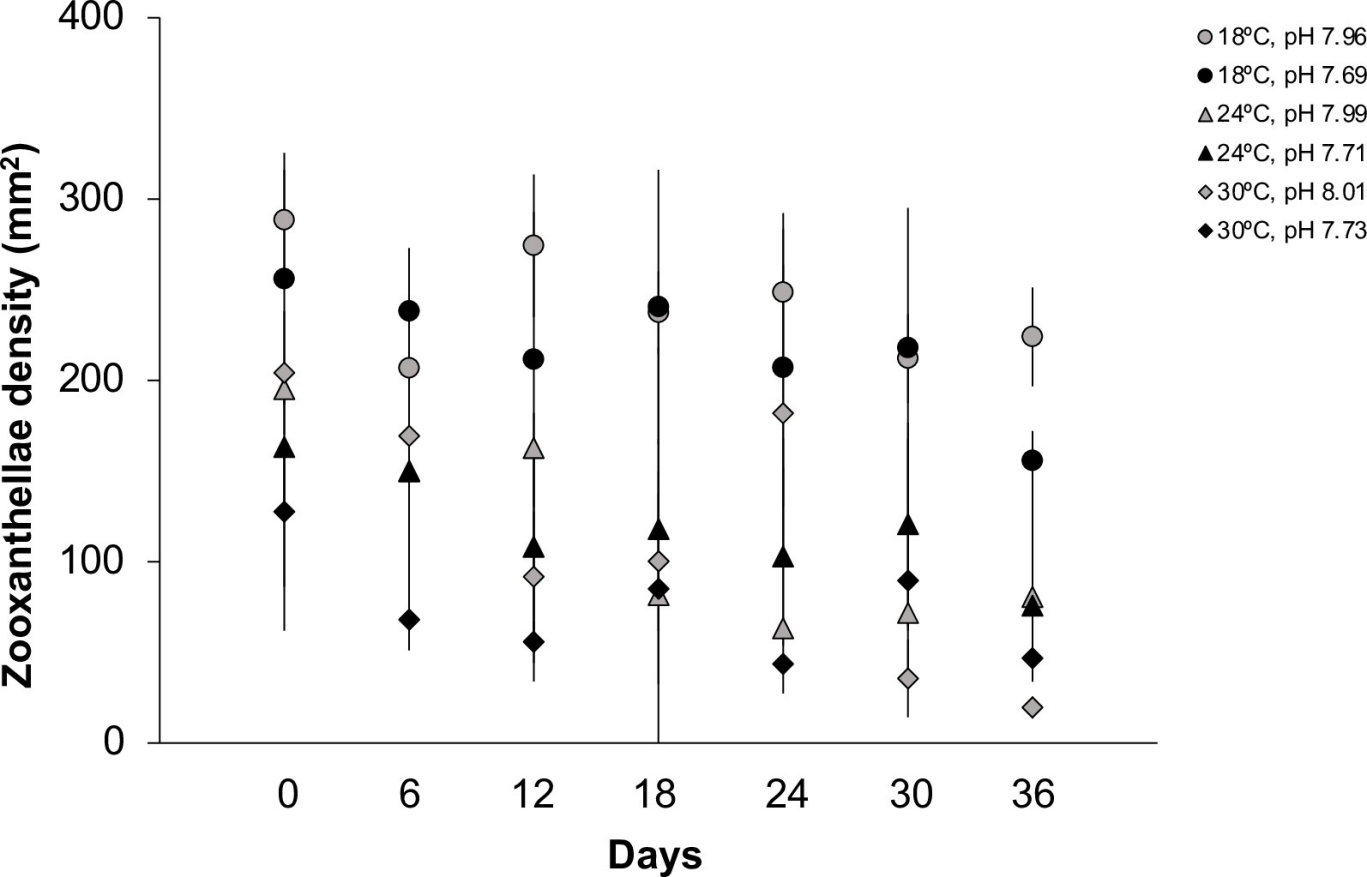
Zooxanthellae density (mm^2^) within the polyps of *Cotylorhiza tuberculata* under different temperatures (18, 24 and 30°C) and pH levels (ambient and future). Circle for 18°C, triangle for 24°C and rhombus for 30°C. Grey for ambient pH and black for future pH. Error bars show standard deviation (SD).

All polyps, buds, and ephyrae from every tested treatment contained symbionts within their tissues (S3 Fig: in S1 File). However, the density of symbionts decreased over time (*p* = 0.0404) in all tested treatments (Fig 7 and Table 6). Temperature (*p* = 0.6012) and pH (*p* = 0.9140), as well as the combination of both variables (*p* = 0.9707) did not significantly affect zooxanthellae density inside *C*. *tuberculata* polyps.

**Table 6:** Summary of results of the linear mixed-models analysis of the zooxanthellae density (mm^2^) (n = 122).

Zooxanthellae density	*df*	X^2^	*P*
Temperature	2	1.0175	0.6012
pH	1	0.0116	0.9140
Temperature × pH	2	0.0593	0.9707
Day	1	4.1992	0.0404*

Chisq = X^2^, *df* = degrees of freedom.

*p < 0.05, *** *p* < 0.001.

## Discussion

Polyps of the jellyfish *C*. *tuberculata* can cope well with the tested temperatures and pH values, as survival and asexual reproduction by budding was unaffected by elevated *P*_CO2_ and temperature in seawater. However, our data suggest that future summer conditions may negatively influence the ability of polyps to release healthy ephyrae, but more targeted analysis is needed. Furthermore, our work reveals that the number of statoliths is not affected by pH or temperature and that bigger statoliths will be formed at elevated temperatures. Finally, we found that the density of zooxanthellae in *C*. *tuberculata* tissues was not impacted by experimental treatment conditions.

### Asexual reproduction by budding

Scyphozoan jellyfish have a complex bipartite life history with alternation of sexual and asexual generations. The size of the adult medusae population and hence the capacity to form blooms is directly determined by the survival, growth, and recruitment of the early life stages (planula larvae, polyp, and ephyrae) [22]. The medusa stage of *C*. *tuberculata* occurs in spring-summer, whereas the benthic polyp stage last for the rest of the year [46]. The health and survival of juveniles and early life stages determine the size of the adult populations of many planktonic species [22]. Therefore, the mechanisms controlling asexual propagation and strobilation of jellyfish polyps, as well as ephyrae recruitment, are important factors to understand jellyfish population dynamics [19, 22].

Asexual reproduction rates of polyps generally increase with increasing temperatures [23, 58]. But when temperature exceeds the natural levels, polyps may decrease or cease to produce buds [59] and may die [23]. In our experiment, all *C*. *tuberculata* polyps survived and reproduced prolifically by budding under simulated current and future (2100) [4] winter scenarios (18°C, pH 7.94 and 7.69), thereby indicating that this life stage can cope with a high CO_2_ (P_CO2_ ~ 1000 ppm) ocean and thrive in future temperate (18°C) winters (Fig 3A). Under mimicked summer scenarios (T2), *C*. *tuberculata* polyps may survive under tested temperatures and pH conditions, but the prolonged exposure of scyphistomae to experimental conditions (*p* < 0.001) and the density of polyps (*p* = 0.002) may negatively affect asexual reproduction by budding and polyp mortality. At 24°C the percentage of mortality was higher under low pH conditions than under the ambient pH treatment (Fig 3E). However, at elevated temperatures (30°C) cumulative percentage of mortality was similar at the end of the experiment under both pH conditions (Fig 3F). Therefore, indicating that, *C*. *tuberculata* polyps will still reproduce asexually by budding even under the most pessimistic conditions considered (30°C and pH 7.73).

The influence of pH on asexual reproduction by budding was negligible (*p* = 0.297), indicating that the OA scenario expected under the RCP8.5 will not lead to massive disappearance of polyps, at least under tested temperatures (18, 24 and, 30°C). This response coincides with previous findings [15, 33, 60, 61]. Winans & Purcell [19] observed no effect of acidification (pH 7.2) on asexual reproduction of polyps of the scyphozoan jellyfish *A*. *labiata*, although in their experiment pH was manipulated by acids and bases addition. This procedure does not accurately mimic the effects of OA because acids decrease water alkalinity but, does not alter inorganic carbon concentrations [62]. Similarly, the growth of polyps of *C*. *capillata* and *Chrysaora hysoscella* were not affected by high *P*_CO2_ levels (pH 7.9) [60]. High tolerance to elevated *P*_CO2_ has also been described for *A*. *aurita* polyps [33, 61], which survived even to extreme acidification conditions (pH 4.5) [33]. In contrast, a decrease in the asexual reproduction by budding under future temperatures and pH levels (pH 7.6) has been reported in the Irukandji jellyfish *A*. nr *mordens* and *C*. *barnesi* [24, 30].

### Strobilation

In nature, strobilation of *C*. *tuberculata* takes place in spring, induced by a seasonal increase of temperature from winter to summer [41, 46, 63]. When summer arrives, *C*. *tuberculata* population survives by means of the medusa stage [41, 46]. In our experiment, strobilation took place at 18°C, corresponding to winter–early spring temperatures in the field. However, the overall percentage of strobilation was higher in the ambient pH treatment than in the high *P*_CO2_ conditions (Fig 3G), suggesting that the capacity of polyps to undergo strobilation might be reduced in the future as a consequence of water acidification. Elevated *P*_CO2_ may eventually affect asexual reproduction in some jellyfish species, as CO_2_ diffuses rapidly into marine organisms causing toxic effects [64–66]. However, asexual reproduction of *C*. *tuberculata* polyps appears to be unaffected by OA under 18°C. Contrary, under simulated summer temperatures (24°C and 30°C) strobilation was very low (Fig 3H and 3I). Prieto et al. [46] also reported a lower strobilation of *C*. *tuberculata* polyps at 21°C than at 19°C.

An increase in temperature is expected to trigger strobilation of *C*. *tuberculata* polyps [43, 46]. However, this phenomenon is more associated with the specific patterns of seasonal temperature transition rather than the absolute change of this variable [46, 63]. It has been recently investigated that certain proteins may act as temperature-dependent “timer” and become highly upregulated by temperature before the onset of strobilation [67]. Polyps may record the duration of the low-temperature period of winter and trigger the metamorphosis process if the activation threshold has been reached [67]. If winters become warmer in the future and seasonal thermal oscillations tend to be softened, a warmer winter such as the one mimicked here (18°C), could reduce or inhibit the strobilation, even if an increase in temperature occurs in summer (24°C and 30°C).

### Ephyrae and statolith formation

Ephyrae from the projected summer conditions suffered malformations, especially those from the ‘RCP8.5 pH only’ scenario. Ephyrae were smaller under acidification conditions at both temperatures but specially at 30°C (Table 4). Our results suggest an independent effect of tested variables on ephyrae diameter. An increase in temperature may lead to the production of bigger ephyrae, however, under future pH levels ephyrae will be smaller and their development may be negatively affected by acidification. However, these findings are based on few numbers of individuals whose ages ranged from 1 to 3 days, and therefore must be interpreted with caution.

Further studies in a larger number of samples would be required to better disentangle the influence of warming and OA on statolith formation by *C*. *tuberculata*, since strobilation was low under summer conditions and not all the replicates were represented in the analyses. Despite the limitations mentioned above, our results suggest that future climate change summer conditions would not lead to a reduction in the number of statoliths synthesized by well-formed ephyrae released from 24 and 30°C and both pH treatments (Fig 5A and Table 4). Nevertheless, the volume of the crystals did not vary in response to low pH or the combination of pH and temperature (Table 4) but, the calculated volume of statoliths was significantly bigger at elevated temperatures (30°C) (Fig 5B and Table 4). This result could be related to the bigger size of ephyrae released at 30°C (Fig 4). The lack of arms, well developed rhopalium and statoliths inside the statocysts would compromise medusae development, equilibrium, and swimming activity. Statoliths change in position alters the stimulation of cilia on the touch plate allowing medusae to orient with respect to gravity [68], the lack of statoliths inside the organ rhopalium might reduce the sensitivity of the sensory cells of the statocyst in the early ephyrae development, probably resulting in swimming alterations. Natural or experimental reduction in the number of statoliths has been shown to produce swimming abnormalities on ephyrae [69]. Furthermore, reduced swimming behavior and smaller size of ephyrae under low pH conditions were reported in *Aurelia* sp. ephyrae [31, 35]. Thus, this points out that a decrease in the survival of *C*. *tuberculata* populations in the future may be proposed as a consequence of reduced ephyrae recruitment caused by the negative effects of acidification on their size and formation, by generating unviable ephyrae.

### Symbiosis with zooxanthellae

*C*. *tuberculata* conserved their zooxanthellae throughout the experiment regardless of tested pH and temperature conditions (Fig 7) thus, suggesting that asexual reproduction of polyps and strobilation was not affected by the lack of symbionts, which are essential for this process to proceed [41]. However, the density of symbionts decreased with the duration of the experiment (*p* < 0.001), particularly at the end of the trial 2. Despite no significant effects of pH nor temperature has been revealed (Table 6), the distribution pattern of zooxanthellae within the polyps varied in the low pH treatments (24°C and 30°C). Also, malformed ephyrae released at the end of the summer experiments (24°C pH 7.71, 30°C pH 8.01 and 7.73) presented fewer zooxanthellae than normal ephyrae (24°C pH 7.96) and an irregular distribution pattern of algae (Fig 6). Effects of acidification on holobionts cnidarians seem to be species-dependent [37] and different effects in the density of symbionts under low pH have been reported in corals and anemones [70, 71]. Nevertheless, polyps of the scyphozoan *Cassiopea* sp. reproduce prolifically under acidification (pH 7.6) and, their tissues mitigate the synergetic effects of hypoxia and elevated *p*CO_2_ conditions by reducing photorespiration and maintaining oxygen homeostasis [72]. Thus, the fitness of zooxanthellate jellyfish polyps as those from *C*. *tuberculata* may be unaffected by low pH conditions and elevated seawater temperatures predicted to occur at the end of the century.

### Adaptation to future ocean conditions

Accurately predicting how jellyfish will respond to future conditions requires the identification of the natural habitat of polyps and the vulnerability of this habitat to climate change. Coastal waters are characterized by greater physicochemical variability than open ocean due to anthropogenic and environmental factors [73, 74]. Shallow coastal waters are highly influenced by large daily and seasonal *P*_CO2_ fluctuations due to photosynthesis and tidal dynamics [75] besides temperature and salinity fluctuations. These changing environmental conditions may influence the phenotypical plasticity of jellyfish polyps to make them more tolerant of climate change drivers and, consequently ensure potential adaptation to future conditions [76, 77]. The range of tolerance of scyphozoans to changing environmental conditions is strongly dependent on species-specific tolerance limits [33] which in turn is determined by the life stage [78]. The asexual reproduction by budding of *C*. *tuberculata* polyps under projected future temperature and pH scenarios evidences the wide physiological limits of the polyp stage of this species, suggesting that polyp populations might survive in a future acidified and warmer Mediterranean, at least under tested conditions. However, if winter temperatures become softened and low temperature levels required to enable the strobilation process are not reached, the percentage of polyp strobilation will be reduced or at least modified in the future. And ephyrae recruitment and fitness will be negatively affected by climate change. Also, the effects of high temperature and low pH on ephyrae size as well as, the malformation of released ephyrae indicate that the pelagic stage is less likely to thrive in the future.

## Conclusions

*C*. *tuberculata* polyps have survived under experimental conditions tested to the future temperature and *P*_CO2_ levels projected to occur by 2100 (30°C and pH 7.7). The evidenced wide tolerance to changing environmental conditions of *C*. *tuberculata* polyps would probably allow this species to undergo a gradual acclimation in the long term to future increases in temperature and OA. The acclimation and adaptability will be probably enhanced by the presence of zooxanthellae within their tissues that might reduce the negative effects of lowering pH. In contrast, future warmer and more acidic conditions are likely to affect the phase transition from polyp to ephyrae and the formation of well-developed ephyrae of *C*. *tuberculata*.

## Supporting information

S1 File Supporting figures and tables. This PDF file contains (1) S1 Fig. Pre-experiment design. (2) S1 Table. Pre-experiment mean physic-chemical parameters for each treatment. (3) S2 Fig. Relationship between ephyrae diameter (*n* = 13) and number or size of statoliths. (4) S3 Fig. Zooxanthellae within the polyps of *Cotylorhiza tuberculata* at the end of Experiment 2.
(PDF)

S2 File Asexual reproduction data. This file contains the data of the results of the number of *C*. *tuberculata* polyps counted at different times and within the different experimental treatments.
(XLSX)

S3 File Ephyrae and statolith data. This file contains the data of *C*. *tuberculata* ephyrae measurements and statolith measurements at different experimental treatments.
(XLSX)

S4 File Zooxanthellae data. This file contains the data of the results of the number of zooxanthellae inside *C*. *tuberculata* polyps counted at different times and within the different treatments.
(XLSX)
