# Supplementary material for: Impact of ocean warming and ocean acidification on asexual reproduction and statolith formation of the symbiotic jellyfish Cotylorhiza tuberculata
Source: PLoS One. 2021 Aug 4;16(8):e0254983. doi: 10.1371/journal.pone.0254983 (PMC8336884; doi:10.1371/journal.pone.0254983)
Supplement: S1 File — This PDF file contains (1) S1 Fig. Pre-experiment design. (2) S1 Table. Pre-experiment mean physic-chemical parameters for each treatment. (3) S2 Fig. Relationship between ephyrae diameter (n = 13) and number or size of statoliths. (4) S3 Fig. Zooxanthellae within the polyps of Cotylorhiza tuberculata at the end of Experiment 2. (PDF) [file pone.0254983.s001.pdf]

S1 File

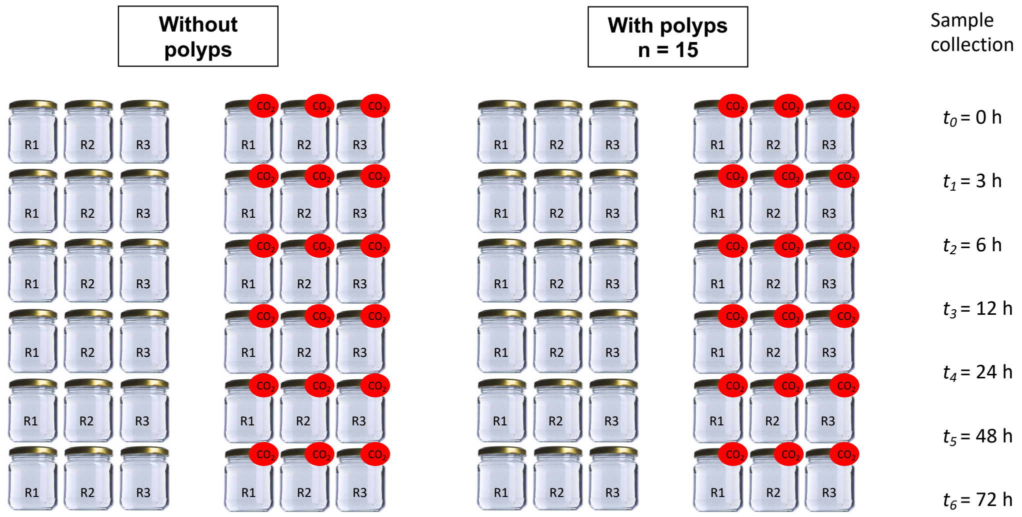

S1 Fig. Pre-experiment design. Seawater from 48 glass flasks with polyps and 48 glass flasks without polyps were used at each temperature treatment (18°C, 24°C and 30°C). Of these 48 glass flasks, 24 were bubbled with current ambient air and 24 bubbled with a gas mixture containing 1000 ppm of CO_2. Sample collection for biogeochemical analysis of three replicate glass flask was done at the beginning of the pre-experiment (t_0), and after 6 h (t_1), 12 h (t_2), 24 h (t_3), 48 h (t_4) and 72 h (t_5) of the pre-experiment commencing.

S1 Table. Pre-experiment mean physic-chemical parameters for each treatment. Temperature (T), pH (pH_{T25}), dissolved oxygen (DO), total alkalinity (A_T) and P_{CO2} in the six experimental treatments. Sample collection of three replicate glass flask was done at the beginning of the pre-experiment (t_0), and after 6 h (t_1), 12 h (t_2), 24 h (t_3), 48 h (t_4) and 72 h (t_5) of the pre-experiment commencing.

Treatment	Flasks	Time	T (°C)	pH_{T25}	DO (mg l^{-1})	A_T (µmol kg^{-1})	pCO_2 (µatm)
18°C, ambient pH	Polyps	t_0	18.1	7.92 (0.004)	7.42 (±0.12)	2491.81 (13.11)	535.79 (41.77)
		t_{72}	(0.015)	7.91 (0.002)	7.00 (±0.03)		
	Seawater	t_0		7.92 (0.004)	7.42 (±0.12)		

		t_{72}		7.87 (0.003)	6.94 (±0.21)		
18°C, future pH	Polyps	t_0		7.71 (0.004)	7.59 (±0.13)	2498.9 (11.49)	1084 (91.51)
		t_{72}		7.62 (0.01)	6.72 (±0.06)		
	Seawater	t_0		7.71 (0.004)	7.59 (±0.13)		
		t_{72}		7.63 (0.01)	7.02 (±0.14)		
24°C, ambient pH	Polyps	t_0	24.3 (0.026)	7.99 (0.002)	7.01 (0.12)	2516.81 (13.89)	495.63 (22.20)
		t_{72}		7.88 (0.004)	6.83 (0.07)		
	Seawater	t_0		7.99 (0.002)	7.01 (0.12)		
		t_{72}		7.91 (0.009)	6.79 (0.10)		
24°C, future pH	Polyps	t_0		7.71 (0.001)	7.13 (0.11)	2507.9 (20.49)	1021 (41.82)
		t_{72}		7.68 (0.008)	6.97 (0.17)		
	Seawater	t_0		7.71 (0.001)	7.13 (0.11)		
		t_{72}		7.69 (0.011)	6.88 (0.21)		
30°C, ambient pH	Polyps	t_0	30.2 (0.017)	8.03 (0.005)	6.34 (0.08)	2509.21 (16.07)	499.12 (15.20)
		t_{72}		7.92 (0.018)	6.20 (0.28)		
	Seawater	t_0		8.03 (0.005)	6.34 (0.08)		
		t_{72}		7.94 (0.031)	6.17 (0.23)		
30°C, future pH	Polyps	t_0		7.86 (0.001)	6.28 (0.09)	2507.9 (20.49)	1003 (77.82)
		t_{72}		7.79 (0.009)	5.93 (0.11)		
	Seawater	t_0		7.86 (0.001)	6.28 (0.09)		
		t_{72}		7.79 (0.009)	5.91 (0.11)		

Mean (SEM) n = 4 per day and treatment.

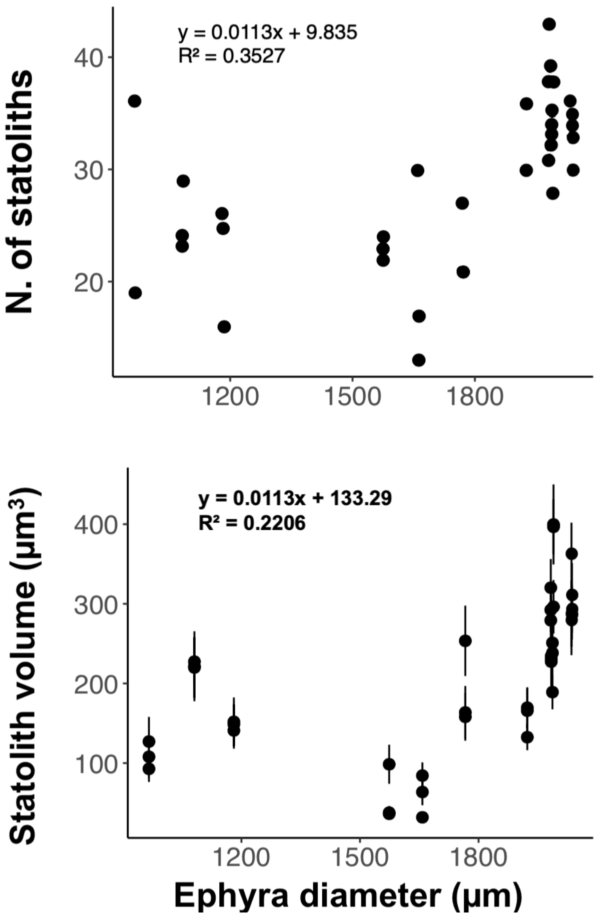

S2 Fig. Relationship between ephyrae diameter (*n* = 13) and number or size of statoliths. Ephyrae diameters represented correspond to well-formed individuals used for statolith analyses that were released at the end of experiment 2.

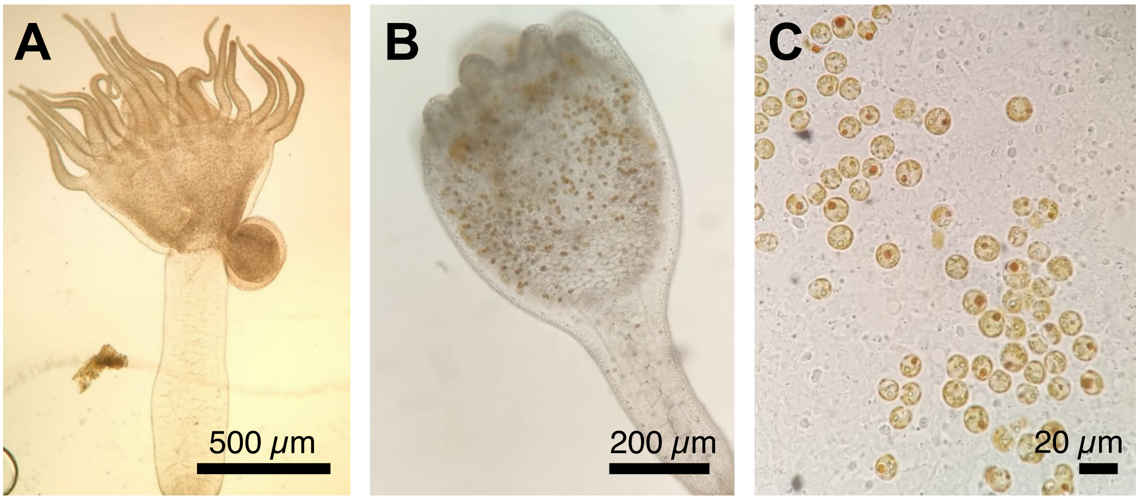

S3 Fig. Zooxanthellae within the polyps of *Cotylorhiza tuberculata* at the end of Experiment 2. A: Mature polyp reproducing asexually by budding, note shaded areas

in the calix and bud evidencing zooxanthellae presence. B: Newly formed polyp with zooxanthellae within the calix. C: Zooxanthellae of the family Symbiodiniceae within the gastrovascular system of a new released ephyrae.
